# Defective NCOA4-dependent ferroptosis in senescent fibroblasts retards diabetic wound healing

**DOI:** 10.1038/s41420-023-01437-7

**Published:** 2023-04-28

**Authors:** Xuerong Wei, Mengqian Liu, Zijun Zheng, Shengxiang Yu, Lei Huang, Jun Ma, Yanbin Gao, Yujie Peng, Lianglong Chen, Rongwei Tan, Zhending She, Lei Yang

**Affiliations:** 1 Department of Burns, Nanfang Hospital, Southern Medical University, Guangzhou, China; 2 Shenzhen Lando Biomaterials Co., Ltd., Shenzhen Engineering Research Center of Implantable Medical Polymer, Guangdong Engineering Research Center of Implantable Medical Polymer, Shenzhen, China

**Keywords:** Trauma, Cell death

## Abstract

Cellular senescence describes a state of permanent proliferative arrest in cells. Studies have demonstrated that diabetes promotes the pathological accumulation of senescent cells, which in turn impairs cell movement and proliferation. Historically, senescence has been perceived to be a detrimental consequence of chronic wound healing. However, the underlying mechanism that causes senescent cells to remain in diabetic wounds is yet to be elucidated. Ferroptosis and ferritinophagy observed in diabetes are due to iron metabolism disorders, which are directly associated with the initiation and progression of diabetes. Herein, we reveal that senescent fibroblasts in diabetic wounds are resistant to ferroptosis and that impaired ferritinophagy may be a contributing cause. Further, the expression of NCOA4, a key factor that influences ferritinophagy, is decreased in both diabetic wound tissue and high glucose-induced senescent fibroblasts. Moreover, NCOA4 overexpression could render senescent fibroblasts more vulnerable to ferroptosis. A faster wound healing process was also linked to the induction of ferroptosis. Thus, resistance to ferroptosis impedes the removal of senescent fibroblasts; promoting ferritinophagy could reverse this process, which may have significant implications for the management of diabetic wounds.

## Introduction

In recent years, the rising prevalence of diabetes has been accompanied by an increase in diabetic ulcers, a serious complication that is the leading cause of disability and amputation in diabetic patients [1, 2]. Hyperglycemia stimulates the formation of advanced glycosylation end products, causes oxidative damage, and induces persistent inflammation, tying diabetes and senescence closely together [3]. Fibroblasts obtained from chronic wounds are susceptible to senescence [4]; however, the molecular and cellular drivers of senescence in chronic non-healing wounds have not been identified, nor has a correlation between their function and poor wound healing outcomes been established.

In diabetic wounds, impaired cellular function and bacterial colonization promote substantial immune cell recruitment and residence, resulting in prolonged, severe local inflammation [5]. This makes diabetic wounds the optimal setting for triggering cellular senescence [6]. For instance, neutrophils generate high levels of reactive oxygen species (ROS), which induce senescence in surrounding cells via the paracrine senescence-associated secretory phenotype (SASP) of neighboring fibroblasts, a process triggered by telomere shortening [7, 8]. Moreover, the expression of cytokines and chemokines is enhanced in diabetic wounds [9]. These essential components of SASP drive macrophages into an inflammatory state [10]. Other local variables, including pathogenic bacterial products [11] and tissue iron [12], may enhance inflammatory responses and impair immune cell function, accelerating the senescence of diabetic wounds. From a clinical standpoint, the prevalence of non-healing diabetic wounds highlights the critical need for new treatment techniques.

Some conditions associated with the formation of senescent cells, such as neurodegeneration (including Alzheimer’s and Parkinson’s disease), osteoarthritis, and idiopathic pulmonary fibrosis, are characterized by a severe iron imbalance, and iron load is commonly correlated with the extent of disease severity [12–14]. In vitro studies have demonstrated that iron accumulates in senescent replicative fibroblasts, and the iron storage protein, ferritin, is abundant in senescent cells [15, 16]. Excessive iron has been suggested to cause cell damage or promote ferroptosis due to redox toxicity [17].

Ferroptosis is a form of programmed cell death initiated by iron-dependent lipid peroxidation [18]. A surplus of active free iron can generate oxidative stress by accelerating the production of ROS through the Fenton reaction [19, 20]. In diabetes, the levels of the primary indicators of iron storage, iron and ferritin, are substantially elevated [21]. Additionally, plasma levels of antioxidant enzymes, such as glutathione (GSH) and SOD, and H_2_O_2_ were lower in diabetic individuals and animal models [22]. Even though intracellular ROS and lipid peroxide (LPO) levels are elevated in diabetic wounds, senescence causes a substantial accumulation of ferritin, which traps free iron and leads to a decline in iron ion levels [12, 23]. Therefore, we hypothesized that senescent cells in diabetic wounds are less vulnerable to ferroptosis. For the breakdown of ferritin and the release of free iron ions, ferritin metabolism is mostly dependent on ferritinophagy, a form of chaperone-mediated autophagy [24]. Notably, autophagy may suppress senescence by enhancing the quality and quantity of proteins and organelles, preserving stemness, fostering genomic stability, or a combination of these mechanisms [25]. Damage to lysosome function in senescent cells reduces ferritinophagy [26], which partially explains the accumulation of iron and ferritin in diabetic wounds.

**Fig. 1:**
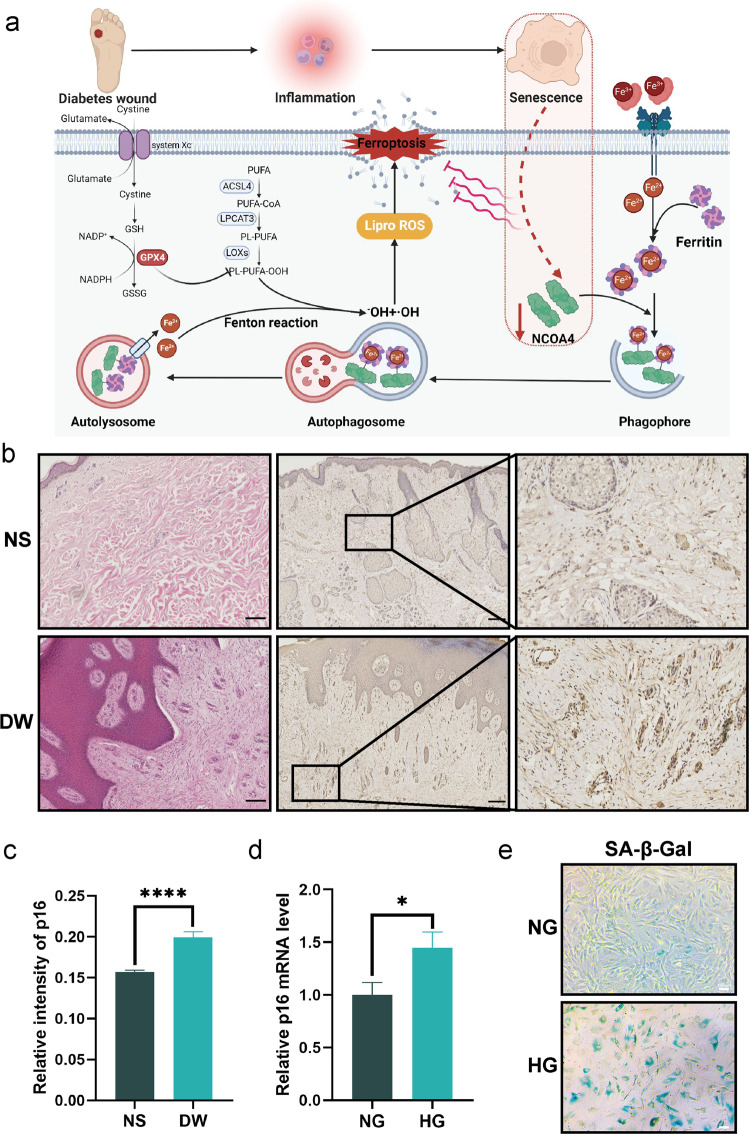
Senescence is linked to diabetic wounds. **a** A hypothesized model of the impact of NCOA4 deficiency in senescent fibroblasts within diabetic wounds. **b** Representative H & E staining and immunostaining of p16 photomicrographs illustrating the architecture of diabetes wounds (DW) and normal skin (NS). Scale bar = 200 μm. **c** Quantification of p16 immunostaining in **b**. **d** The mRNA level of p16 were detected by real-time PCR in normal glucose cultured fibroblasts (NG) and high glucose cultured fibroblasts (SF). **e** SA-β-GAL staining illustrates increased senescent fibroblasts after high glucose culture. Scale bar = 100 μm. Data represent the mean ± SEM of triplicates. *P < 0.05; ****P < 0.0001.

To confirm these hypotheses, we collected tissue samples from diabetic wounds and analyzed ferritinophagy-related indicators to detect clinical evidence of dysregulated iron metabolic homeostasis. We constructed a high glucose-induced senescent cell model and a diabetic mouse model to investigate the impact of senescence on ferritinophagy and ferroptosis. We investigated the role of nuclear receptor coactivator 4 (NCOA4) in regulating ferroptosis (Fig. 1a). Finally, we observed that inducing ferroptosis in diabetic mice promoted wound healing to some extent. Our findings identify novel therapeutic targets for the treatment of diabetic wounds.

## Results

### Senescent cells accumulation in diabetic wounds

Tissue samples from patients with diabetic ulcers were collected and discarded skin flap samples from healthy individuals undergoing plastic surgery were used as the control. Firstly, the tissue was stained with hematoxylin and eosin (H & E). Diabetic wounds (DW) differ from normal skin (NS) in that they have a thicker epidermis, exhibit keratinocyte proliferation, decreased dermal collagen content, and disrupted fibroblast organization (Fig. 1b). Using immunohistochemical staining, we evaluated the senescence phenotype of cells in the diabetic wound samples and discovered that the protein expression of p16 was increased in this tissue (Fig. 1b, c). Increased senescence-related β galactosidases (SA-β-GAL) was also confirmed in diabetic wounds (Supplementary Fig. S1). As the most common cell type in the skin, fibroblasts serve as both synthesis cells, depositing extracellular mesenchyme, and signaling cells, secreting growth factors that are crucial for intercellular signaling and healing [27, 28]. Due to the fact that fibroblasts are essential for wound healing and tissue repair, we selected fibroblasts as the in vitro study’s subject. Then we cultivated primary mouse skin fibroblasts in a complete medium with a high glucose content of 33.3 mM, while the glucose concentration in the control group was 5.56 mM, to investigate whether a high glucose environment can cause fibroblast senescence in vitro. After 14 days of high glucose therapy, the high glucose (HG) group had considerably more cells at the same passage stained with SA-β-GAL than the control group (normal glucose, NG) (Fig. 1e). These findings were supported by qPCR analysis, wherein the mRNA expression of p16 was significantly elevated in diabetic wounds (Fig. 1d). Then, we evaluated the impact of high glucose on cell migration. The wound healing assay showed that cells cultured in HG displayed a lower migratory capacity compared with cells in NG medium (Supplementary Fig. S2). This data indicates that the high glucose environment in diabetic wounds causes cellular senescence and suggests a connection between senescence and pathological repair.

### Senescence renders fibroblasts immune to ferroptosis

**Fig. 2:**
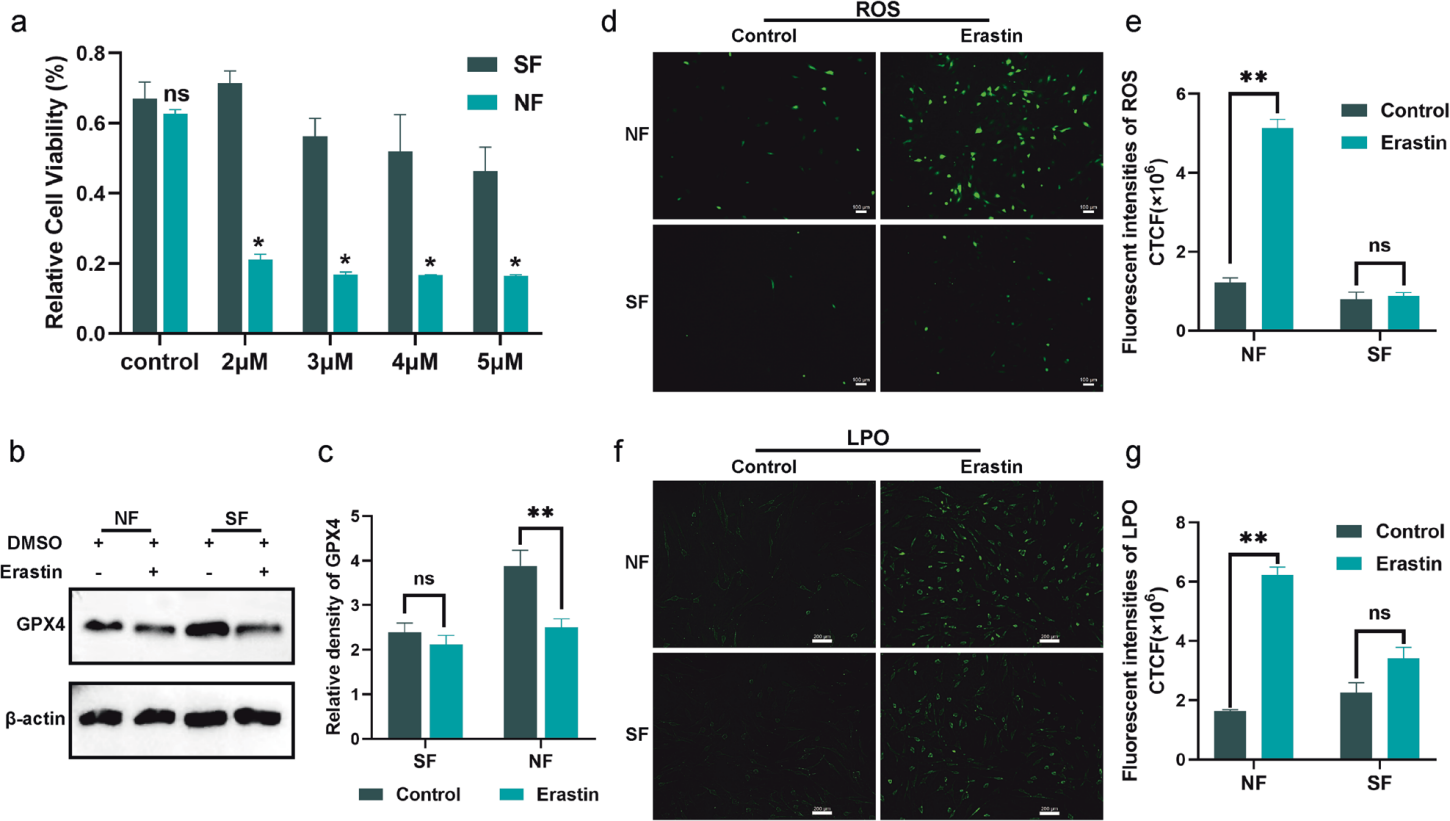
Senescent fibroblasts exhibit a ferroptosis-resistant phenotype. **a** Relative cell viability of erastin (2, 3, 4, 5 μΜ) -stimulated fibroblasts. **b** Protein level of GPX4 after 5 μΜ erastin treatment. **c** Quantification of GPX4 immunoblotting in **b**. **d**, **f** Intracellular ROS and LPO level in fibroblasts with the indicated treatment. Scale bar in **d** = 200 μm, Scale bar in **f** = 100 μm. **e**, **g** Quantification of ROS and LPO level in **d** and **f**. Data represent the mean ± SEM of triplicates. *P < 0.05; **P < 0.01; ns nonsignificant, NF normal fibroblast, SF senescent fibroblast, CTCF corrected total cell fluorescence.

Different doses of erastin, a traditional inducer of ferroptosis [29], were administered to normal fibroblasts (NF) and senescent fibroblasts (SF) resulting from high glucose, to test the sensitivity of senescence to ferroptosis. Following 24 hours of treatment with various concentrations of erastin (2, 3, 4, and 5 μm), a CCK8 assay showed that the activity of SF was much higher than that of NF, with no differences in the control treatment (Fig. 2a). In ferroptosis, glutathione peroxidase 4 (GPX4) is a crucial checkpoint [30]. Lipid peroxide damage and ferroptosis result from an accumulation of lipid peroxides in glutathione-inhibited cells when GPX4 activity is low [31]. After 6 h of ferroptosis-induction treatment (erastin concentration of 5μm) in SF and NF, GPX4 expression in senescent cells was not significantly different to that in the control group; however, GPX4 expression was drastically lower in NF (Fig. 2b, c). We then evaluated the fluctuation in peroxide concentration in response to this treatment using several approaches. The ROS content of NF that were treated with erastin were significantly elevated, whereas no significant changes were evident in SF (Fig. 2d, e). In line with ROS data, the LPO concentration in SF was not considerably affected (Fig. 2f, g). This series of experiments indicates that high glucose-induced senescence renders fibroblasts immune to ferroptosis and may be responsible for the significant accumulation of senescent cells in diabetic wounds.

### Altered content of ferritin and free iron in senescence

**Fig. 3:**
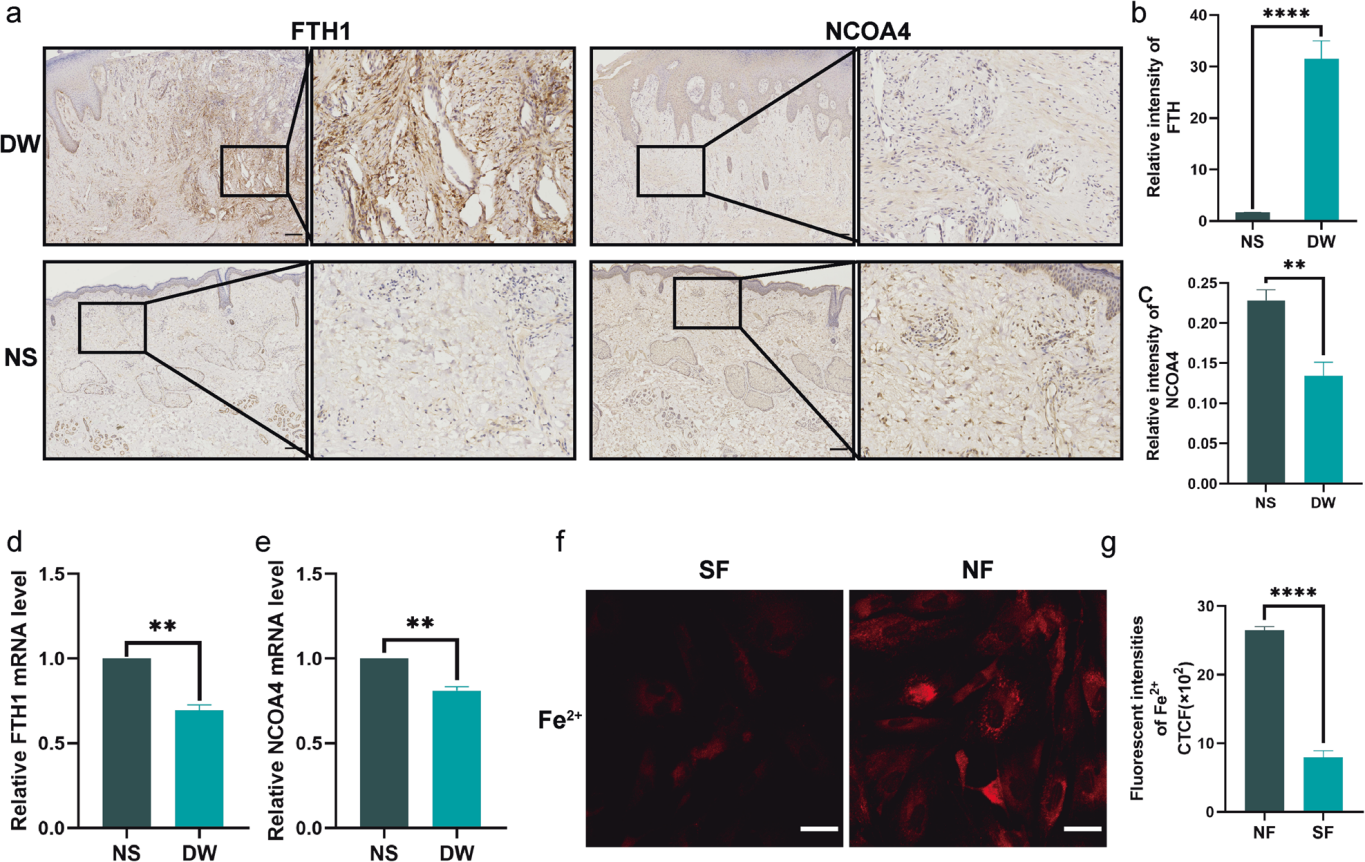
Diabetes wounds exhibit impaired ferritin degradation. **a** Immunohistochemistry staining of FTH1 and NCOA4 in DW and NS. Scale bar = 200 mm. **b**, **c** Quantification of FTH1 and NCOA4 immunostaining in **a**. **d**, **e** The mRNA level of FTH1 and NCOA4 were detected by real-time PCR in DW and NS. **f** Intracellular free iron ions in fibroblasts. Scale bar = 50 μm. **g** Quantification of intracellular free iron ions in **f**. Data represent the mean ± SEM of triplicates. **P < 0.01; ****P < 0.0001. DW diabetes wounds, NS normal skin, NF normal fibroblast, SF senescent fibroblast, CTCF corrected total cell fluorescence.

The underlying cause for senescence rendering cells resistant to ferroptosis was then investigated. Changes in the amount of ferritin and stored iron ions are a consequence of senescence-induced iron metabolism disorders [32]. Immunohistochemical staining demonstrated that ferritin expression in diabetic wound tissue was substantially higher than in normal skin tissue (Fig. 3a, b). Ferritin is mostly degraded via ferritinophagy, where it is carried to lysosomes to be degraded and to liberate ferric ions, thus preserving intracellular iron homeostasis [33]. It has been proposed that senescence hinders the ferritinophagy process and that the inability to breakdown ferritin results in a decrease in free intracellular iron ions [34]. Thus, we have investigated the role of NCOA4 in this process, as it is a cargo receptor that facilitates the entry of ferritin into the lysosome and is necessary for the maintenance of iron homeostasis [24]. Immunohistochemical staining revealed that the expression of NOCA4 was considerably reduced in diabetic wounds (Fig. 3a, c). Furthermore, qPCR confirmed that the mRNA expression of NCOA4 was reduced in diabetic wound tissues (Fig. 3e). In contrast to the change in its protein expression, mRNA expression of FTH1 was decreased, as ferritin expression is regulated at the transcriptional and translational levels (Fig. 3d). Additionally, after regulation, changes at the protein level of ferritin precede changes at the RNA level [35], thus providing evidence that ferritin accumulation in the context of aging is unlikely to be regulated by the transcription or translation of ferritin. Using the fluorescent dye FerroOrange, we also measured intracellular free iron ions, which were significantly (*P* < 0.0001) lower in high glucose-induced SF than in NF (Fig. 3f, g). Therefore, senescence-induced ferritin buildup is not caused by an elevation in ferritin mRNA transcription, but rather by a decrease in NCOA4 expression, resulting in impaired ferritinophagy.

### Ferritinophagy impairment in senescent fibroblasts

**Fig. 4:**
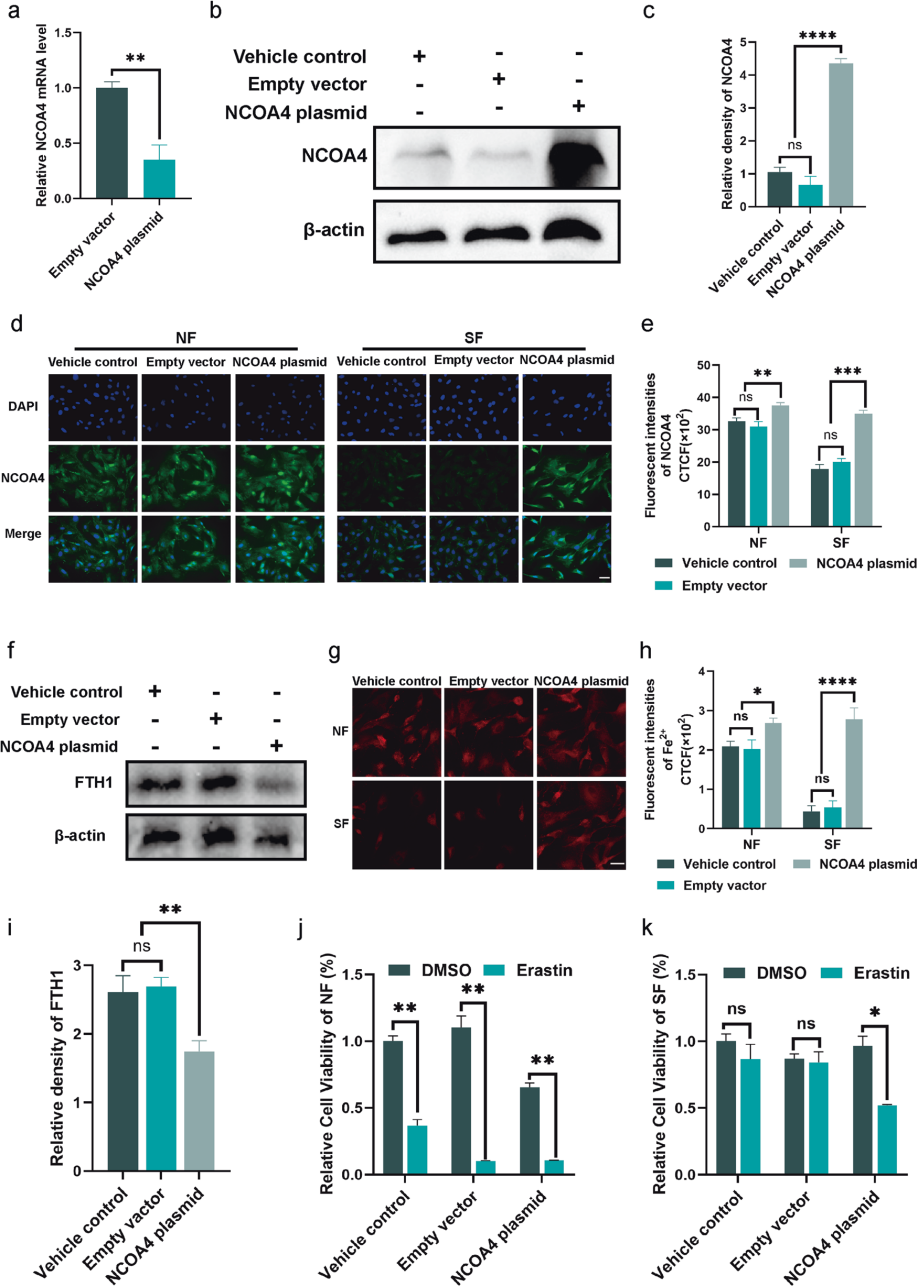
Overexpression of NCOA4 repaired the ferritinophagy in senescent fibroblast. **a**, **b** The mRNA and protein level of NCOA4 in fibroblasts to detect the transfection efficiency of NCOA4. **c** Quantification of NCOA4 immunoblotting in **b**. **d** Immunofluorescence staining of NCOA4 in NF and SF after transfected NCOA4 plasmid. Scale bar = 100 μm. **e** Quantification of NCOA4 immunofluorescence staining in **d**. **f** Immunoblot of FTH1 in SF with the indicated treatment. **i** Quantification of FTH1 immunoblotting in **f**. **g** Intracellular free iron ions in fibroblasts with the indicated treatment. Scale bar = 50 μm. **h** Quantification of intracellular free iron ions in **g**. **j**, **k** Relative cell viability of NF and SF with erastin (5 μΜ) stimulation after transfection. Data represent the mean ± SEM of triplicates. **P < 0.01; ****P < 0.0001. DW diabetes wounds, NS normal skin, NF normal fibroblast, SF senescent fibroblast, CTCF corrected total cell fluorescence.

We investigated whether altered NOCA4 expression was associated with the insensitivity of senescence to ferroptosis. To enhance intracellular NCOA4 expression, we created an overexpression plasmid and transfected it into primary mouse skin fibroblasts. Both qPCR and western blot indicated that the expression of both mRNA and protein of NCOA4 was enhanced after transfection, demonstrating successful plasmid transfection (Fig. 4a, b). According to previous experimental findings, protein and mRNA expression of NCOA4 reduced in diabetic wounds (Fig. 3a, c). Therefore, we hypothesized that this effect is owing to a senescent phenotype. After successfully transfecting fibroblasts with NCOA4 plasmid, normal and high glucose cultures were added, and immunofluorescence staining was used to evaluate NCOA4 expression. In fibroblasts treated with high glucose, NCOA4 expression was much lower than that in cells cultivated in normal media. The transfection of the NCOA4 plasmid improved NCOA4 expression in the high glucose group (Fig. 4d, e). NCOA4-mediated ferritinophagy is primarily responsible for ferritin breakdown. A decrease in FTH1 expression and an increase in free ferric ions were observed in SF with an overexpression of NCOA4 (Fig. 4f, g). This demonstrates that the intracellular ferritin and ferric ions in senescent cells can be altered by modulating the expression of NCOA4.

Given that overexpression of NCOA4 increased the iron content of senescent cells, we anticipated that it may also overcome the resistance of senescent cells to ferroptosis. The CCK8 assay demonstrated that erastin dramatically decreased the cellular activity of normal glucose-cultured cells, regardless of NCOA4 overexpression. In contrast, erastin diminished cellular viability exclusively in senescent cells that were cultivated in high glucose when NCOA4 was overexpressed (Fig. 4j, k). Consistent with CCK8 results, erastin induced an increase in LPO formation exclusively in senescent cells that overexpressed NCOA4 (Supplementary Fig. S3).

These findings suggest that a high glucose microenvironment reduces NCOA4 expression in SF. Overexpression of NCOA4 by plasmid transfection accelerates the breakdown of ferritin, raises the concentration of ferric ions, and increases the susceptibility of SF to ferroptosis. Therefore, the insensitivity of SF to ferroptosis may be correlated with a decrease in NCOA4 expression.

### Inducing ferroptosis to promote diabetic wound healing

**Fig. 5:**
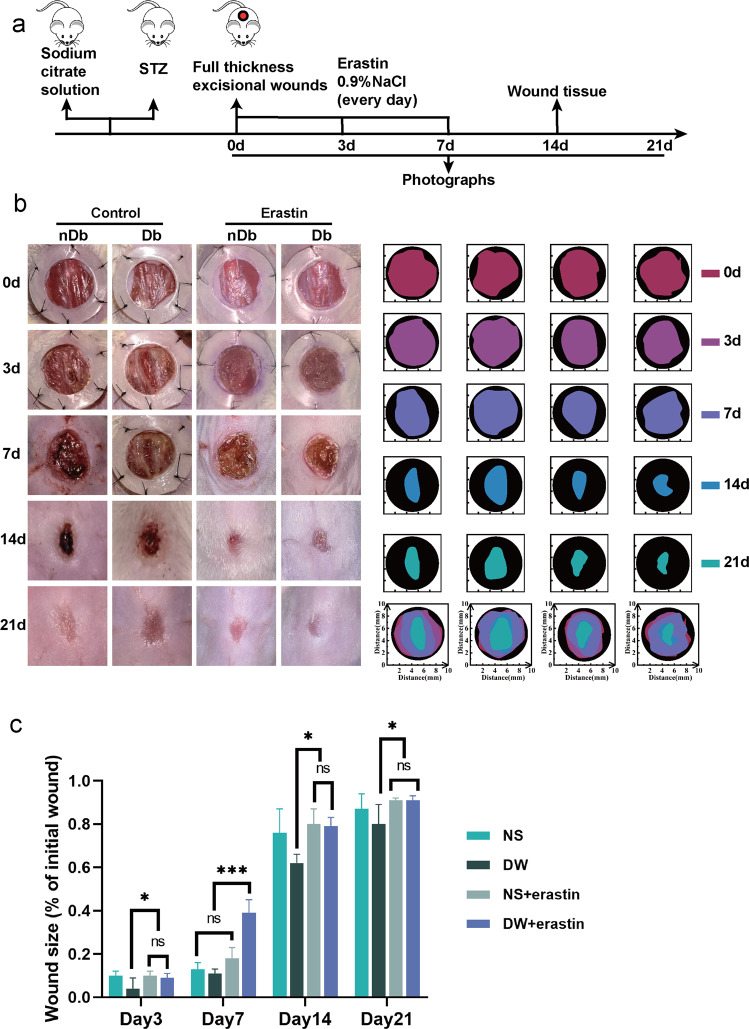
Inducing ferroptosis increases increases the rate of wound healing in vivo. **a** A schematic view of the experimental setting. **b** Photographs of wound closure and simulation plots of wound closure. **c** The quantitative analysis of wound closure. Data represent the mean ± SEM of triplicates. *P < 0.05; ***P < 0.001; ns nonsignificant. STZ streptozotocin; nDb non-diabetic, Db diabetic.

**Fig. 6:**
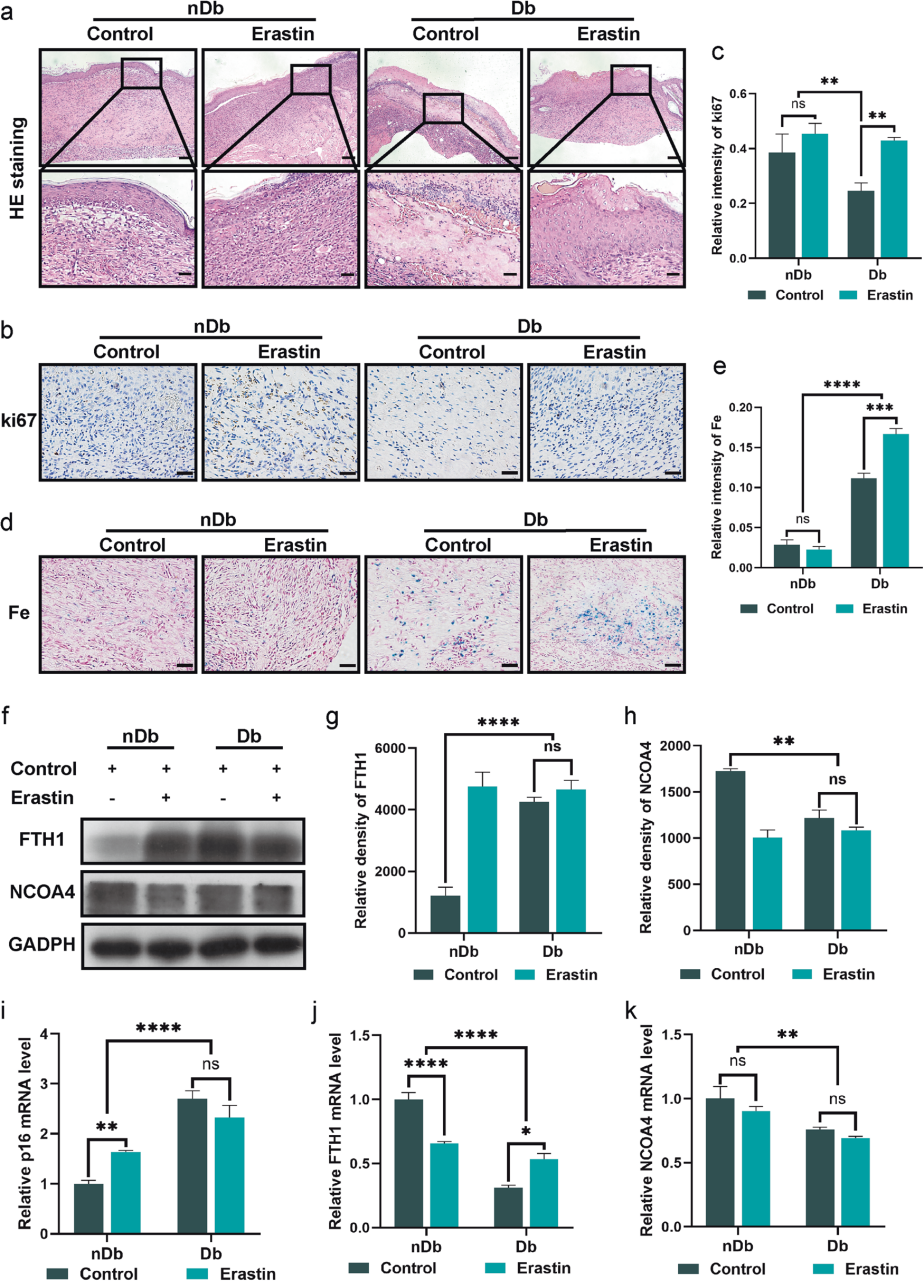
Erastin accelerate wounds healing process. **a** H&E staining of the wound indicated the healing situation on day 14. Scale bar = 200 μm (upper). **b** Immunohistochemistry staining of Ki67 in nDb and Db wound sections at day 14 after surgery. Scale bar = 50 μm. **c** Quantitative evaluation of the Ki67-positive cell ratio. **d** Prussian blue staining of the nDb and Db wounds at day 14 after surgery. Scale bar = 50 μm. **e** Quantitative evaluation of the Prussian blue-positive cell ratio. **f** Immunoblot of FTH1 and NCOA4 in wounds with the indicated treatment. **g**, **h** Quantification of FTH1 and NCOA4 immunoblotting in **f**. **i**, **j** The mRNA level of p16, FTH1, and NCOA4 were detected by real-time PCR in wounds with the indicated treatment. Data represent the mean ± SEM of triplicates. **P < 0.01; ***P < 0.001; ****P < 0.0001; ns nonsignificant, nDb non-diabetic, Db diabetic.

We demonstrated the effects of variation in ferritinophagy on diabetic wounds as well as establish the relationship between diabetic wounds and ferroptosis in vivo. Full-thickness skin defect surgery was performed on the backs of both BALB/c mice with streptozotocin (STZ)-induced type 1 diabetes mice and non-diabetic mice using sodium citrate buffer. The wound site was treated with erastin for seven consecutive days following surgery to induce ferroptosis; photographs were taken on days 0, 3, 7, 14, and 21; and tissue was collected on day 14 (Fig. 5a). Overall, wound healing in diabetic mice was found to be slower than that in non-diabetic mice, and the erastin intervention accelerated wound healing in diabetic wounds to a rate consistent with normal acute wound healing (Fig. 5b, c). On the 14^th^ day postoperatively, we performed H & E staining on the wounds which revealed that saline-treated non-diabetic wounds had completed epithelialization, whereas the diabetic wounds had only reached the granulation stage and were significantly infiltrated by inflammatory cells. The non-diabetic wounds treated with erastin exhibited clear epithelization, and the diabetic wounds treated with erastin commenced the epithelization process at a significantly greater rate than that in the control group (Fig. 6a). Furthermore, ki67 staining indicated that erastin therapy increased cellular proliferation in mice with diabetic wounds, to a level equivalent to that in non-diabetic mice wounds (Fig. 6b, c).

To further elucidate the effects of ferritinophagy in vivo, we analyzed the levels of FTH and NCOA4 in wound tissue. We discovered that ferritin expression was higher in diabetic wounds, and that therapy with erastin decreased FTH expression in non-diabetic wounds, however, erastin had no impact on diabetic wounds (Fig. 6f, g). Evidently, NCOA4 expression was low in diabetic wounds, and erastin treatment had a minimal effect on NCOA4 expression, which is consistent with previous reports involving human tissue (Fig. 6f, h). Similarly, we examined the expression of FTH1 and NCOA4 at the genetic level. We found that the mRNA expression of FTH1 and NCOA4 was both higher in non-diabetic wounds than in diabetic wounds (Fig. 6j, k). This also corroborates the results obtained from clinical samples. We then quantified the iron content of the wounds. The iron content of diabetic wounds was shown to be greater than that of non-diabetic wounds; however, erastin had no effect on the accumulation of iron ions in diabetic wounds (Fig. 6d, e).

The rate of wound healing is lower in diabetic mice than in non-diabetic mice, and the effects of an altered ferritinophagy process is consistent with in vitro test results. Furthermore, treatment with erastin aided in the healing of diabetic and non-diabetic wounds but had no effect on ferritinophagy.

## Discussion

The established link between senescence and chronic, non-healing diabetic wounds, has led to senescence being a proposed therapeutic target [36]. Despite the lack of well-established research on the connection between diabetic wounds and senescence, a growing number of publications are contributing to a deeper understanding of the role of senescence in diabetic wounds [37]. Firstly, senescent cells create a collection of SASPs that regulate the nearby microenvironment, thereby influencing various aspects of wound regeneration, including cell proliferation, matrix remodeling, and angiogenesis [38]. Another factor that hinders the healing of diabetic wounds is the accumulation of oxidative damage. In normal circumstances, inflammatory signals elicit an immune response that results in the elimination of abnormal cells [39]. However, in aged tissue, these clearance systems are disturbed, resulting in an intense immune response, aseptic inflammation, and consequent prolongation of pathological processes [40]. Furthermore, the conversion of M1 macrophages into M2 macrophages is inhibited, and their diminished capacity renders them insusceptible to migration to sites where senescent cells congregate [41]. Thus, the overall primary properties of senescent cells, including SASP, ROS, and malfunction of the immune system, inhibit diabetic wound healing. Consequently, targeting senescent cells in diabetic chronic wounds may be useful prospective therapy. Several potential treatments are already in development to either eliminate senescent cells or minimize their negative effects to accelerate diabetic wound healing. Zhao et al. described a nanomaterial that can target senescent cells and induce apoptosis, demonstrating that it could be used to promote diabetic wound healing [42].

Since ferroptosis was identified, many emerging studies have been focused on the prevention and treatment of tumors [18]. Owing to our expanding understanding of ferroptosis, its role in diabetic wounds has attracted considerable interest in recent years [43]. In ferroptosis and diabetes, depleted GPX4 and GSH, increased iron, and enhanced lipid peroxidation are common characteristics [44]. There is a large body of research attributing some diabetic complications to high glucose levels that promote a shift in the upstream core regulating components, leading to iron overload and a reduction in antioxidant capacity via distinct pathways [45, 46]. Current research suggests that autophagy regulates ferroptosis through modulating cellular iron homeostasis and generating reactive oxygen species [47]. Ferroptosis and ferritinophagy are observed in diabetic complications due to inherent cell metabolic pathway disorders, such as iron overload induced by faulty iron metabolism, which is directly associated to the formation and progression of diabetic complications [48].

It has been shown that senescent fibroblasts induced by other stimuli (radiation, replicative and oncogenic) are highly resistant to ferroptosis [34]. Congruent with this finding, our research shows that ferritin significantly accumulates and NCOA4 expression is drastically decreased in senescent fibroblasts induced by a high glucose medium. Under these conditions, ferritin binds to intracellular iron, reducing the available free iron. Thus, cells develop a resistance to ferroptosis owing to the reduction in free iron.

In addition to the radiation, replicative and oncogenic involved in that study, high glucose is also a common stimulus that induces cellular senescence. Hyperglycemia is a proximal trigger for many downstream molecular disorders in diabetes, including cellular senescence. The excessive production of reactive oxygen species (ROS) and the extensive oxidative attack on multicellular targets, including mitochondrial structures, establish a perpetuating cycle [49, 50]. We believe it is plausible to hypothesize that senescence explains why high glucose-induced cells are resistant to ferroptosis and have altered iron homeostasis protein expression. And this hyper-resistance to ferroptosis is considered to be one of the reasons behind the high rate of senescent cells in diabetic wounds.

Future research should concentrate on targeting the pathophysiological aspects of senescence and mitigating its negative effects on diabetic wound healing. Our study offers preliminary evidence that therapy with ferroptosis-inducers accelerate the healing of diabetic wounds. Due to the inherent complexity of the wound environment, the possibility that erastin promotes diabetic wound healing by other processes cannot be precluded. For example, erastin can promote diabetic wound healing by inhibiting the membrane transporter SLC7A11 to activate efferocytosis [51]. Therefore, targeting the induction of senescent cell death and achieving precise management in the context of complicated wounds is a promising direction. Overall, our findings provide strong foundations for the future development of therapeutics to target ferritinophagy and eliminate senescence in diabetic wounds.

## Materials and methods

### Human specimen acquisition

In this study, non-diabetic patients (*n* = 3) who underwent surgery for diverse reasons, such as flap grafting following accidental trauma, were included as the healthy control group. Patients with diabetic foot ulcers (*n* = 3) who underwent ulcer excision surgery were also included as the test group. They offered adequate wound and peri-wound tissue for examination. This research was authorized by the Ethics Committee of the Nanfang Hospital of Southern Medical University.

### Primary fibroblast harvest and treatment

As reported previously, primary fibroblasts were isolated from the skin of neonatal BALB/c mice [52]. Then primary fibroblasts were cultivated in a complete medium with a high glucose content of 33.3 mM (HG) for 14 days, and the glucose concentration in the control group was 5.56 mM (NG). Medium changes were performed at two-day intervals until the cells were ready for testing. All tests were conducted on cells between passages three and five.

### ROS assay

Total intracellular ROS levels were measured using the ROS Assay Kit (Beyotime, S0033, China) according to the manufacturer’s instructions. After high glucose treatment for 14 days, HG group and NG group fibroblasts of the same passage (P5/6) were seeded at a density of 1 × 10^5^ cells/well in 24-well cell culture plate and treated with erastin at the concentration of 5 μm for 6 h. Cells were incubated with 10 mM DCFH-DA reagent for 20 min at 37 °C according to the manufacturer’s instructions. Finally, cells were observed using an Olympus IX73 fluorescence microscope.

### LPO assay

The cell preparation is same as ROS assay. After treatment, cells were incubated with Liperfluo (Dojindo, L248, China) for one hour according to the manufacturer’s instructions. Then the cells were washed with PBS and observed using an Olympus IX73 fluorescence microscope (Olympus, Tokyo, Japan).

### Fe^2+^evaluation

Briefly, fibroblasts (P2) were transfected with either a control plasmid or NOCA4 overexpression plasmid, then the cultures were treated with HG and NG. After 14 days’ culture, FerroOrange (Dojindo, F374, China) was applied to reveal the distribution and expression of ferrous iron in the cytoplasm of living fibroblasts (P5/6). The fibroblasts culture medium was replaced with serum-free medium containing 1 μM FerroOrange, and then cells were incubated at 37 °C for 30 min before being detected using a confocal fluorescence microscope (Zeiss, LSM980, Oberkochen, Germany).

### Western blotting

Total cell and tissue proteins were extracted. Bicinchoninic acid method was used to determine the protein concentration and quantify the total amount of protein. SDS-polyacrylamide gel electrophoresis was performed to separate the protein extracts. After the electrophoresis, the gel was transferred to polyvinylidene fluoride membrane. The membranes were incubated with 5% skim milk for 1 h. The primary antibodies: NCOA4 (Sino Biological, #203674, China, 1:400), FTH (Abcam, #ab75972, USA, 1:500), GPX4 (Abcam, #ab125066, USA, 1: 1000); GAPDH (Abcam, #ab181602, USA, 1:1000); β-actin (Abcam, #ab8226, USA, 1:500) were added to incubate with the membranes at 4 °C overnight. Secondary antibody was incubated for 1 h at room temperature. Densitometry analysis was performed using the ImageJ software.

### Cell transfection with NCOA4 overexpression plasmid

Primary fibroblasts were transfected with either a control plasmid or NOCA4 overexpression plasmid (both sourced from VectorBuilder, Guangzhou, China) using UltraCruz® Transfection Reagent (Santa Cruz Biotechnology, sc-395739, Texas, USA) according to the manufacturer’s guidelines. After adding the plasmid and transfection reagent for 6 h, the medium was changed to a HG or NG medium for further studies. The efficacy of transfeciton was analyzed by western blot and qPCR 48 h after transfection.

### Mouse diabetes model

Care and treatment of animals were performed in accordance with institutional procedures as well as national laws and rules. The Experimental Animal Ethics Committee of the Nanfang Hospital authorized the experimental procedure. Diabetic (Db) and non-diabetic (nDb) groups (*n* ≥ 3) were randomly formed in male BALB/c mice aged six weeks. The diabetic group received a sodium citrate solution of STZ (50 mg/kg) through intraperitoneal injection for five days, and the control group received sodium citrate solution.

### Histological analysis

SA-β-Gal staining, p16, NCOA4, FTH1 immunohistochemistry, Perl’s staining and H & E staining were performed in wound tissue (day 14) of human and mice. (see Supplementary Materials and Methods).

### Statistical analysis

All experiments were from at least 3 individual substances and repeated at least three times unless otherwise indicated. Error bars represent SEM. Student’s t-test was used to compare two groups of independent samples. For multiple-group comparisons, we used one-way ANOVA as a parametric method. Statistical significance was defined by *P* value (*P < 0.05, **P < 0.01, ***P < 0.001, ****P < 0.0001) and the *P* values were two-sided. *P* < 0.05 was considered statistically significant. NS indicates no significant difference. All statistical analyses were performed with GraphPad Prism (version 8.0, GraphPad Software, SanDiego, CA).

## Supplementary information

SUPPLEMENTARY MATERIALS

## Data Availability

The data sets that support the findings of the current study are available from the corresponding authors upon reasonable request.

## References

[1] Jeffcoate WJ, Vileikyte L, Boyko EJ, Armstrong DG, Boulton AJM (2018). Current challenges and opportunities in the prevention and management of diabetic foot ulcers. Diabetes Care.

[2] Armstrong DG, Boulton AJM, Bus SA (2017). Diabetic foot ulcers and their recurrence. N Engl J Med.

[3] Wilkinson HN, Hardman MJ (2022). Cellular senescence in acute and chronic wound repair. Cold Spring Harb Perspect Biol.

[4] Wilkinson HN, Clowes C, Banyard KL, Matteuci P, Mace KA, Hardman MJ (2019). Elevated local senescence in diabetic wound healing is linked to pathological repair via CXCR2. J Invest Dermatol.

[5] Louiselle AE, Niemiec SM, Zgheib C, Liechty KW (2021). Macrophage polarization and diabetic wound healing. Transl Res.

[6] Furman D, Chang J, Lartigue L, Bolen CR, Haddad F, Gaudilliere B (2017). Expression of specific inflammasome gene modules stratifies older individuals into two extreme clinical and immunological states. Nat Med.

[7] Sampson MJ, Winterbone MS, Hughes JC, Dozio N, Hughes DA (2006). Monocyte telomere shortening and oxidative DNA damage in type 2 diabetes. Diabetes Care.

[8] Elks CE, Scott RA (2014). The long and short of telomere length and diabetes. Diabetes.

[9] Shanmugam N, Reddy MA, Guha M, Natarajan R (2003). High glucose-induced expression of proinflammatory cytokine and chemokine genes in monocytic cells. Diabetes.

[10] Prattichizzo F, De Nigris V, Mancuso E, Spiga R, Giuliani A, Matacchione G (2018). Short-term sustained hyperglycaemia fosters an archetypal senescence-associated secretory phenotype in endothelial cells and macrophages. Redox Biol.

[11] Bana B, Cabreiro F (2019). The microbiome and aging. Annu Rev Genet.

[12] Zeidan RS, Han SM, Leeuwenburgh C, Xiao R (2021). Iron homeostasis and organismal aging. Ageing Res Rev.

[13] Zucca FA, Segura-Aguilar J, Ferrari E, Munoz P, Paris I, Sulzer D (2017). Interactions of iron, dopamine and neuromelanin pathways in brain aging and Parkinson’s disease. Prog Neurobiol.

[14] Smith MJ, Fowler M, Naftalin RJ, Siow RCM (2020). UVA irradiation increases ferrous iron release from human skin fibroblast and endothelial cell ferritin: consequences for cell senescence and aging. Free Radic Biol Med.

[15] Killilea DW, Atamna H, Liao C, Ames BN (2003). Iron accumulation during cellular senescence in human fibroblasts in vitro. Antioxid Redox Signal.

[16] DeRuisseau KC, Park YM, DeRuisseau LR, Cowley PM, Fazen CH, Doyle RP (2013). Aging-related changes in the iron status of skeletal muscle. Exp Gerontol.

[17] Park E, Chung SW (2019). ROS-mediated autophagy increases intracellular iron levels and ferroptosis by ferritin and transferrin receptor regulation. Cell Death Dis.

[18] Dixon SJ, Lemberg KM, Lamprecht MR, Skouta R, Zaitsev EM, Gleason CE (2012). Ferroptosis: an iron-dependent form of nonapoptotic cell death. Cell.

[19] Zhou RP, Chen Y, Wei X, Yu B, Xiong ZG, Lu C (2020). Novel insights into ferroptosis: implications for age-related diseases. Theranostics.

[20] Jomova K, Valko M (2011). Advances in metal-induced oxidative stress and human disease. Toxicology.

[21] Simcox JA, McClain DA (2013). Iron and diabetes risk. Cell Metab.

[22] Azevedo-Martins AK, Lortz S, Lenzen S, Curi R, Eizirik DL, Tiedge M (2003). Improvement of the mitochondrial antioxidant defense status prevents cytokine-induced nuclear factor-kappaB activation in insulin-producing cells. Diabetes.

[23] Hughes CE, Coody TK, Jeong MY, Berg JA, Winge DR, Hughes AL (2020). Cysteine toxicity drives age-related mitochondrial decline by altering iron homeostasis. Cell.

[24] Mancias JD, Wang X, Gygi SP, Harper JW, Kimmelman AC (2014). Quantitative proteomics identifies NCOA4 as the cargo receptor mediating ferritinophagy. Nature.

[25] Mizushima N, Levine B (2020). Autophagy in human diseases. N Engl J Med.

[26] Tai H, Wang Z, Gong H, Han X, Zhou J, Wang X (2017). Autophagy impairment with lysosomal and mitochondrial dysfunction is an important characteristic of oxidative stress-induced senescence. Autophagy.

[27] Lynch MD, Watt FM (2018). Fibroblast heterogeneity: implications for human disease. J Clin Invest.

[28] Mascharak S, desJardins-Park HE, Longaker MT (2020). Fibroblast heterogeneity in wound healing: hurdles to clinical translation. Trends Mol Med.

[29] Jiang X, Stockwell BR, Conrad M (2021). Ferroptosis: mechanisms, biology and role in disease. Nat Rev Mol Cell Biol.

[30] Yang WS, SriRamaratnam R, Welsch ME, Shimada K, Skouta R, Viswanathan VS (2014). Regulation of ferroptotic cancer cell death by GPX4. Cell.

[31] Friedmann Angeli JP, Schneider M, Proneth B, Tyurina YY, Tyurin VA, Hammond VJ (2014). Inactivation of the ferroptosis regulator Gpx4 triggers acute renal failure in mice. Nat Cell Biol.

[32] Sato T, Shapiro JS, Chang HC, Miller RA, Ardehali H (2022). Aging is associated with increased brain iron through cortex-derived hepcidin expression. Elife.

[33] Hou W, Xie Y, Song X, Sun X, Lotze MT, Zeh HJ (2016). Autophagy promotes ferroptosis by degradation of ferritin. Autophagy.

[34] Masaldan S, Clatworthy SAS, Gamell C, Meggyesy PM, Rigopoulos AT, Haupt S (2018). Iron accumulation in senescent cells is coupled with impaired ferritinophagy and inhibition of ferroptosis. Redox Biol.

[35] Fuhrmann DC, Mondorf A, Beifuss J, Jung M, Brune B (2020). Hypoxia inhibits ferritinophagy, increases mitochondrial ferritin, and protects from ferroptosis. Redox Biol.

[36] Palmer AK, Gustafson B, Kirkland JL, Smith U (2019). Cellular senescence: at the nexus between ageing and diabetes. Diabetologia.

[37] Wilkinson HN, Hardman MJ (2021). Wound senescence: a functional link between diabetes and ageing?. Exp Dermatol.

[38] Coppe JP, Patil CK, Rodier F, Sun Y, Munoz DP, Goldstein J (2008). Senescence-associated secretory phenotypes reveal cell-nonautonomous functions of oncogenic RAS and the p53 tumor suppressor. PLoS Biol.

[39] Parrinello S, Samper E, Krtolica A, Goldstein J, Melov S, Campisi J (2003). Oxygen sensitivity severely limits the replicative lifespan of murine fibroblasts. Nat Cell Biol.

[40] Duan J, Duan J, Zhang Z, Tong T (2005). Irreversible cellular senescence induced by prolonged exposure to H2O2 involves DNA-damage-and-repair genes and telomere shortening. Int J Biochem Cell Biol.

[41] Bannon P, Wood S, Restivo T, Campbell L, Hardman MJ, Mace KA (2013). Diabetes induces stable intrinsic changes to myeloid cells that contribute to chronic inflammation during wound healing in mice. Dis Model Mech.

[42] Zhao R, Jin X, Li A, Xu B, Shen Y, Wang W (2022). Precise diabetic wound therapy: PLS nanospheres eliminate senescent cells via DPP4 targeting and PARP1 activation. Adv Sci (Weinh).

[43] He J, Li Z, Xia P, Shi A, FuChen X, Zhang J (2022). Ferroptosis and ferritinophagy in diabetes complications. Mol Metab.

[44] Altamura S, Kopf S, Schmidt J, Mudder K, da Silva AR, Nawroth P (2017). Uncoupled iron homeostasis in type 2 diabetes mellitus. J Mol Med (Berl).

[45] Wu Y, Zhao Y, Yang HZ, Wang YJ, Chen Y (2021). HMGB1 regulates ferroptosis through Nrf2 pathway in mesangial cells in response to high glucose. Biosci Rep.

[46] Cundy T, Holden A, Stallworthy E (2021). Early worsening of diabetic nephropathy in type 2 diabetes after rapid improvement in chronic severe hyperglycemia. Diabetes Care.

[47] Zhou B, Liu J, Kang R, Klionsky DJ, Kroemer G, Tang D (2020). Ferroptosis is a type of autophagy-dependent cell death. Semin Cancer Biol.

[48] Venkatesan P, Varghese J, Arthi TS, James JV, Anura A, Prasad J (2021). Evidence of dysregulated iron homeostasis in newly diagnosed diabetics, but not in pre-diabetics. J Diabetes Complications.

[49] Wang Q, Nie L, Zhao P, Zhou X, Ding Y, Chen Q (2021). Diabetes fuels periodontal lesions via GLUT1-driven macrophage inflammaging. Int J Oral Sci.

[50] Bertelli PM, Pedrini E, Hughes D, McDonnell S, Pathak V, Peixoto E (2022). Long term high glucose exposure induces premature senescence in retinal endothelial cells. Front Physiol.

[51] Maschalidi S, Mehrotra P, Keceli BN, De Cleene HKL, Lecomte K, Van der Cruyssen R (2022). Targeting SLC7A11 improves efferocytosis by dendritic cells and wound healing in diabetes. Nature.

[52] Lichti U, Anders J, Yuspa SH (2008). Isolation and short-term culture of primary keratinocytes, hair follicle populations and dermal cells from newborn mice and keratinocytes from adult mice for in vitro analysis and for grafting to immunodeficient mice. Nat Protoc.

