# Supplementary material for: Defective NCOA4-dependent ferroptosis in senescent fibroblasts retards diabetic wound healing
Source: Cell Death Discov. 2023 Apr 28;9:138. doi: 10.1038/s41420-023-01437-7 (PMC10147701; doi:10.1038/s41420-023-01437-7)
Supplement: Supplementary file 1 — SUPPLEMENTARY MATERIALS [file 41420_2023_1437_MOESM1_ESM.pdf]

SUPPLEMENTARY MATERIALS AND METHODS

Animals

Care and treatment of animals were performed in accordance with institutional procedures as well as national laws and rules. The Experimental Animal Ethics Committee of the Nanfang Hospital authorized the experimental procedure. Six-week-old mice (BALB/c) with a body weight of 18 to 23 g were obtained from Southern Medical University Experimental Animal Center (Guangzhou, People's Republic of China). Mice were kept in a carefully regulated environment.

Primary fibroblast harvest

As reported previously, primary fibroblasts were isolated from the skin of neonatal BALB/c mice (Lichti et al., 2008). Mice were culled then submerged in 75% alcohol for five minutes. Dorsal skin (3 cm×5 cm) was separated using sterile ophthalmic scissors, placed in a 10 cm petri dish, rinsed twice with PBS containing 1% penicillin-streptomycin. Fat and blood vessels were removed and PBS was discarded. Five mL of 0.2% collagenase was added to the prepared skins, which were cut into small pieces using scissors. The mixture was incubated for 4 h at 37°C. Subsequently, 30 mL of Dulbecco's Modified Eagle Medium (DMEM) was used to dilute the digest. DMEM was then filtered through a 70 μm cell strainer using a centrifugal tube to remove undigested skin fragments. Following a three-minute centrifugation at 1,000 rpm, the resulting sediment was retained. The sediment was then resuspended in DMEM containing 10% fetal bovine serum and 1% penicillin-streptomycin. After 48 hours of incubation at 37°C and 5% carbon dioxide, the medium and non-adherent cells were extracted and new medium was added. Medium changes were performed at two-day intervals until the cells were ready for testing. All tests were conducted on cells between passages three and five.

Senescence Associated-β-Galactosidase (SA-β-gal) expression assay

A SA-β-Gal staining kit (Abcam, AB102534) was used according to manufacturer instructions to observe the expression of SA-β-Gal in primary skin fibroblasts. The medium for cell culture in the 12-well plate was discarded and washed three times with 1×PBS. The cells were fixed for 10 to 15 minutes at room temperature using Fixative Solution (0.5 mL per well). The Staining Solution Mix was prepared (Staining Solution, 470 μL; Staining Supplement, 5 μL; 20 mg/mL X-Gal in DMF, 25 μL) for each well once the cells had been placed in the Fixative

Solution. Cells were then rinsed with 1×PBS twice, then the Solution Mix was added (0.5mL per well). The plate was covered and sealed with sealing film to prevent CO_2 from entering, then incubated for one hour at 37°C. Cells were examined under a microscope to detect the appearance of a blue hue.

Cell viability assay

The CCK-8 (Dojindo, CK04) was used to measure cell viability. Samples including 10,000 cells per well were inoculated onto 96-well plates for a 24-hour preculture. The indicated stimulus treatment was then added for 6–24 hours. The culture media was replaced with fresh medium containing 10 μL of CCK-8 solution per well and incubated for one hour in a 37 ℃ incubator (Thermo Fisher, USA). The absorbance at 450 nm was measured using a microplate reader (Cytation5 Cell Imaging Multi-Mode Reader, BioTek, USA).

ROS evaluation

Total intracellular ROS levels were measured using the ROS Assay Kit (Beyotime, S0033). The fluorescent DCFH-DA probe was loaded 24 hours after treatment with erastin. After dilution with 1:1000 serum-free media, the final concentration of DCFH-DA was 10 μmol/L. The cell culture medium was removed and the appropriate volume of DCFH-DA was added for adequate dilution. A sufficient volume to adequately cover the cells was added then incubated at 37°C for 20 minutes. The cells were rinsed three times with serum-free cell culture media to eliminate any DCFH-DA that had not yet entered the cells. Finally, cells were observed using an Olympus BX73 fluorescence microscope with excitation and emission wavelengths of 488 and 525 nm, respectively.

LPO evaluation

Liperfluo (Dojindo, L248), a fluorescent probe, was used to detect cellular lipid hydroperoxides. Fibroblasts were dispensed into 24-well plates at a density of 1 x 10^5 cells. After treatment, cells were incubated with Liperfluo (10 μM) for one hour. Then the cells were washed with PBS and observed using an Olympus BX73 fluorescence microscope.

Cell transfection with NCOA4 overexpression plasmid

Primary fibroblasts were transfected with either a control plasmid or NOCA4 overexpression plasmid (both sourced from VectorBuilder, Guangzhou, China) using UltraCruz® Transfection Reagent (Santa Cruz Biotechnology, sc-395739) according to the manufacturer’s guidelines. One microgram of NCOA4

overexpression plasmid and 4 μL of UltraCruz® Transfection Reagent were combined with 150 μL DMEM, without FBS, at room temperature for five minutes. The two solutions were then properly combined and left for 15 minutes at room temperature. The cells on six-well plates were washed three times with PBS, and the culture medium was replaced with fresh DMEM without FBS. Each well was then filled with 300μL of the aforementioned mixture while the plates were shaken. After six hours of incubation at 37°C, the medium was replaced with fresh complete medium, and the cells were treated with erastin for 24 hours.

In vitro wound healing assay

Primary fibroblasts were planted at a density of 6×10^5 cells/well in six-well plates, grown to confluence, and serum-starved overnight. The monolayer was scratched with a 200 μL pipette tip, then the wells were washed with PBS to remove non-adherent cells. The cells were imaged at 0, 24, 48, and 72 hours after being cultivated in serum-free media at 37°C with the indicated reagents. ImageJ 6.0 software was used to measure the wound area. The migration area rate (A%) was calculated using the ratio of wound closure area (A_t) to initial wound area (A_0):

$$A\% = [(A_0 - A_t) / A_0] \times 100\%$$

Quantitative Reverse-Transcription Polymerase Chain Reaction

Using the TRIzol (Leagene, NR0002) reagent, total RNA was extracted from wound tissues. After quantifying the concentration and purity of the RNA samples, reverse transcription was performed using the Evo M-MLV RT Premix (Accurate biology, AG11706) for quantitative polymerase chain reaction (qPCR). Polymerase chain reaction amplification was carried out using a 20 μL reaction mixture including 4 μL of cDNA and 10 μL of SYBR® Green Premix Pro Taq HS qPCR Kit (Rox Plus) (Accurate biology, AG11718). After pre-denaturing at 95°C for 30 seconds, 40 cycles were performed using a two-step PCR reaction procedure (denaturing at 95°C for five seconds, followed by annealing and extension at 60°C for 30 seconds) with QuantStudio 6 (Thermo, America). Comparative threshold cycle (ddCt) analysis was utilized to determine gene expression levels. The relative mRNA levels were validated against the mRNA levels of glyceraldehyde-3-phosphate dehydrogenase (GAPDH). The primer sequences are available in the **Supplementary Table S1**.

Supplementary Table S1. Primer sequences used for RT-qPCR

Primer	Forward Sequence	Reverse Sequence
hp16	CCAACGCACCGAATAGTTAC	GCGCTGCCCATCATCATG
hFTH1	CATCAACCGCCAGATCAACC	CACATCATCGCGGTCAAAGT

hNCOA4	GTGTAGTGATGCACGGAGGG	CCAGGTGACGGCTTATGCAAC
hGAPDH	TGCACCACCAACTGCTTAGC	GGCATGGACTGTGGTCATGAG
mp16	TGAATCTCCGCGAGGAAAGC	TGCCCATCATCATCACCTGAA
mFTH1	CCATCAACCGCCAGATCAAC	GCCACATCATCTCGGTCAAA
mNCOA4	AAGAAAGTGGGAAGCCTCAG	AGATCACAAACTGCTGGGAG
mβ-Actin	AGCCATGTACGTAGCCATCC	GCTGTGGTGGTGAAGCTGTA

Mouse diabetes model

Diabetic (Db) and non-diabetic (nDb) groups were randomly formed in male BALB/c mice aged six weeks. The diabetic group received a sodium citrate solution of STZ (50 mg/kg) through intraperitoneal injection for five days, and the control group received sodium citrate solution. Blood was withdrawn from the tail veins of mice before modeling, three days after STZ injection and once weekly for four consecutive weeks following modeling to measure their random blood glucose levels and body weight. If the blood glucose level of the mice was below 8.9 mmol/L before modeling and above 16.7 mmol/L upon each monitoring after modeling, with considerable weight loss, increased drinking and urine output, and yellowing of body hair, the diabetes model was regarded to have been successfully induced. The blood glucose levels of non-diabetic mice were also measured at the aforementioned times.

In vivo wound healing assessment

The mice were divided into Db and nDb groups and intraperitoneally administered 50 mg/kg of 10% sodium pentobarbital. Depilatory paste was used to depilate the dorsum of mice. Under aseptic conditions, a 10 mm-diameter round, full-thickness excisional wound was created on the back of the mouse using Iris scissors and fixed with a rubber band to prevent the wound from contracting. Wound healing was observed at days 0, 3, 7, 14, and 21, and the corresponding indicators were measured at days 7, 14, and 21, respectively.

Perl's staining

Perl's staining was used to identify cellular iron accumulation. Briefly, tissue sections were immersed for three minutes in distilled water, then incubated in Perl solution (Sigma-Aldrich, USA) for thirty minutes, and then washed three times in PBS. Finally, sections were treated for 2 minutes in 3,3-diaminobenzidine (DAB) and counterstained with hematoxylin.

Immunohistochemistry staining analysis

For immunohistochemistry, formaldehyde-fixed, deparaffinized tissue sections were stained overnight with rabbit polyclonal antibodies against FTH1 (Abcam, # ab75972, 1:500) and NCOA4 (Abmart, # TD4255,1:400) and for 1 h with secondary antibody Goat Anti-Rabbit IgG H&L (HRP) (Abcam, #ab205718, 1:500).

Immunofluorescence staining analysis

The cells were seeded in 24-well plates with 5×10^5 cells per well. After fixation, the cells were treated with 0.2% Triton X-100 for 10 minutes and then sealed with 5% BSA for one hour. The first antibody, NCOA4 (Abmart, # TD4255,1:400), was then incubated overnight at 4°C, and the second antibody was added and incubated for one hour. The cells were washed with PBS and incubated for 10 minutes with DAPI stain at room temperature. The observation and photographs were taken using an Olympus BX73 microscope.

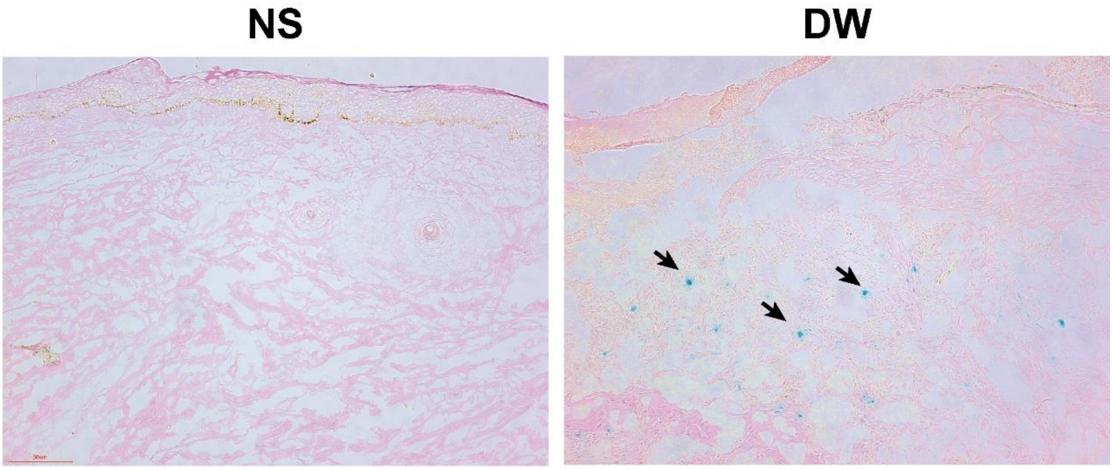

Supplementary Figure S1. SA-β-Gal staining in human normal skin and diabetes wounds. SA-β-GAL staining illustrates increased senescent cells in diabetes wounds. Normal skin (NS), Diabetic wounds (DW). Scale bar = 50 μm.

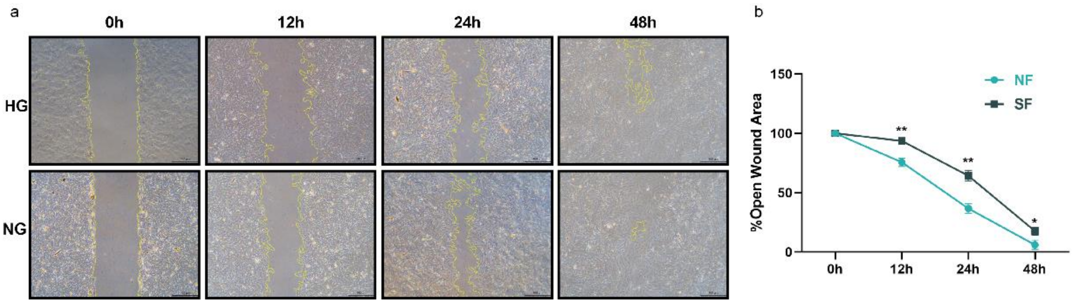

Supplementary Figure S2. Reduced migration capacity of senescent cells induced by high glucose. (a, b) Images and quantification of fibroblasts migration in a wound healing assay. Scale bar = 400 μm. Data represent the mean ± SEM of triplicates. *P < 0.05; **P< 0.01.

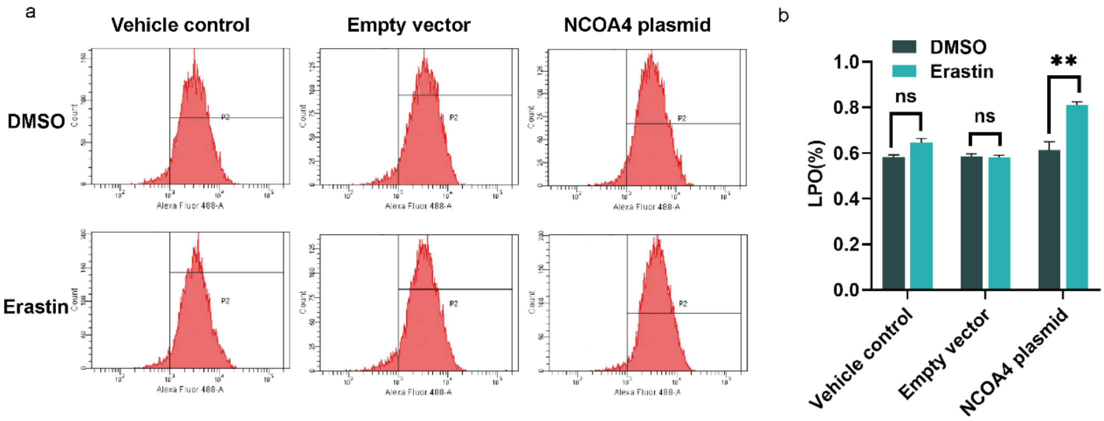

Supplementary Figure S3. NCOA4 overexpression makes senescent fibroblasts susceptible to ferroptosis. (a, b) Representative data of the histogram of Liperfluo fluorescence in senescent fibroblasts with the indicated treatment by flow cytometer. Data represent the mean ± SEM of triplicates. **P< 0.01; ns, nonsignificant.

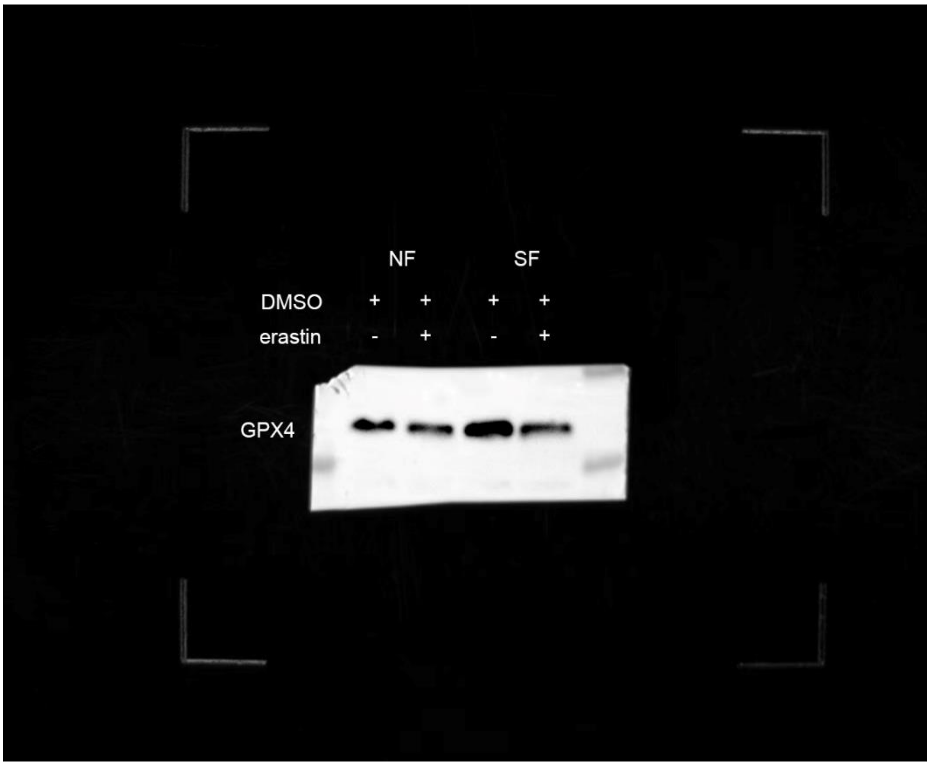

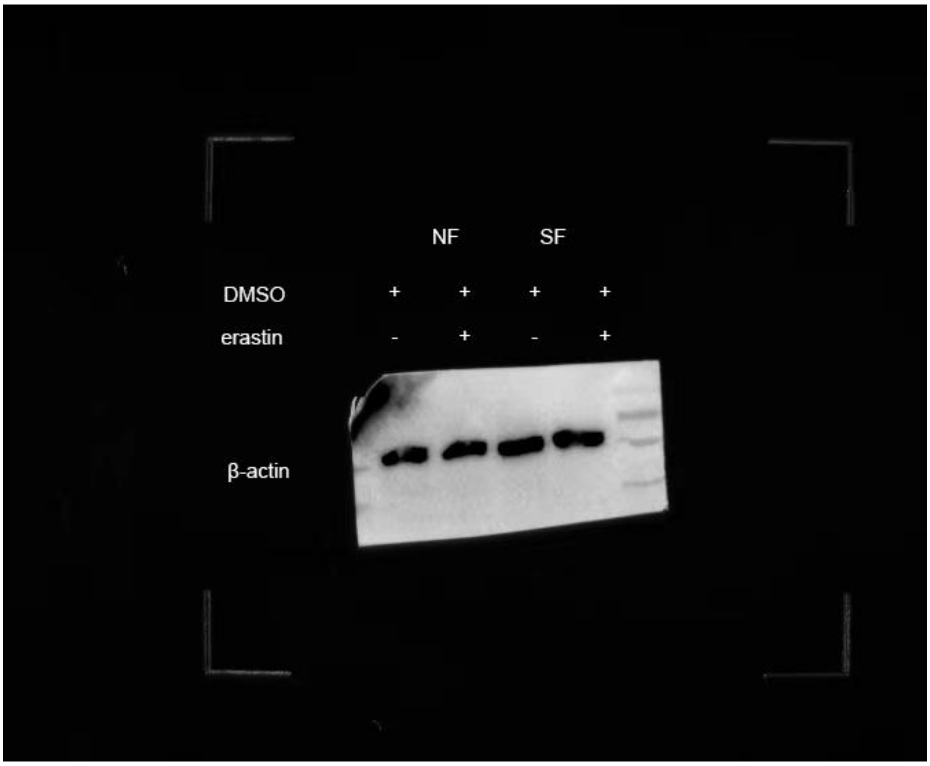

Supplementary Figure S3. Original western blot of Figure2 b.

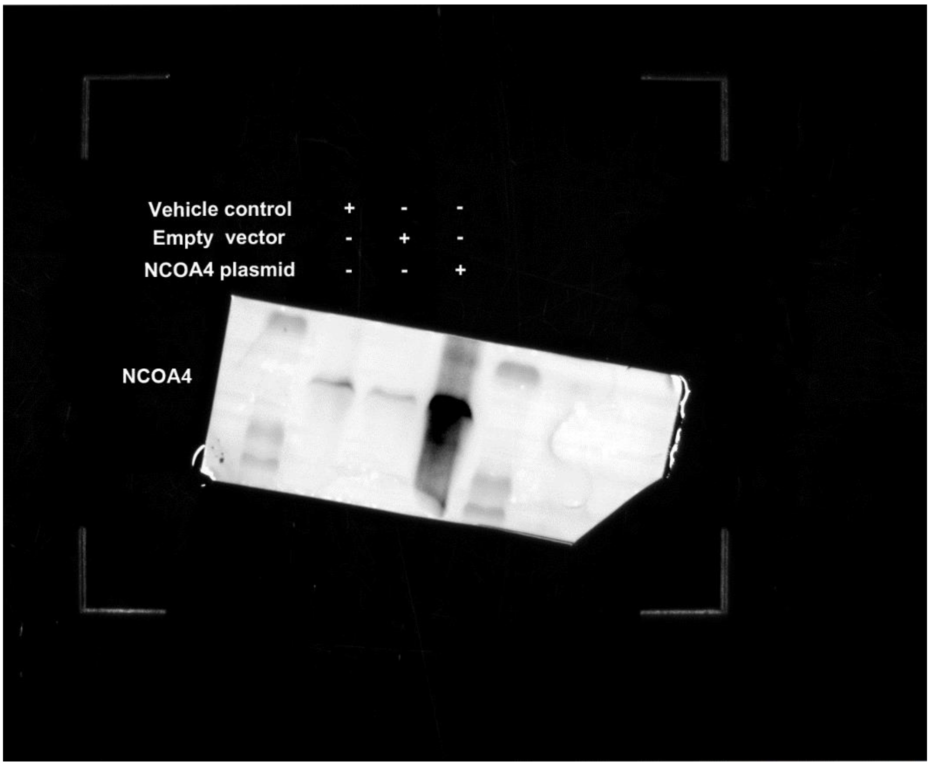

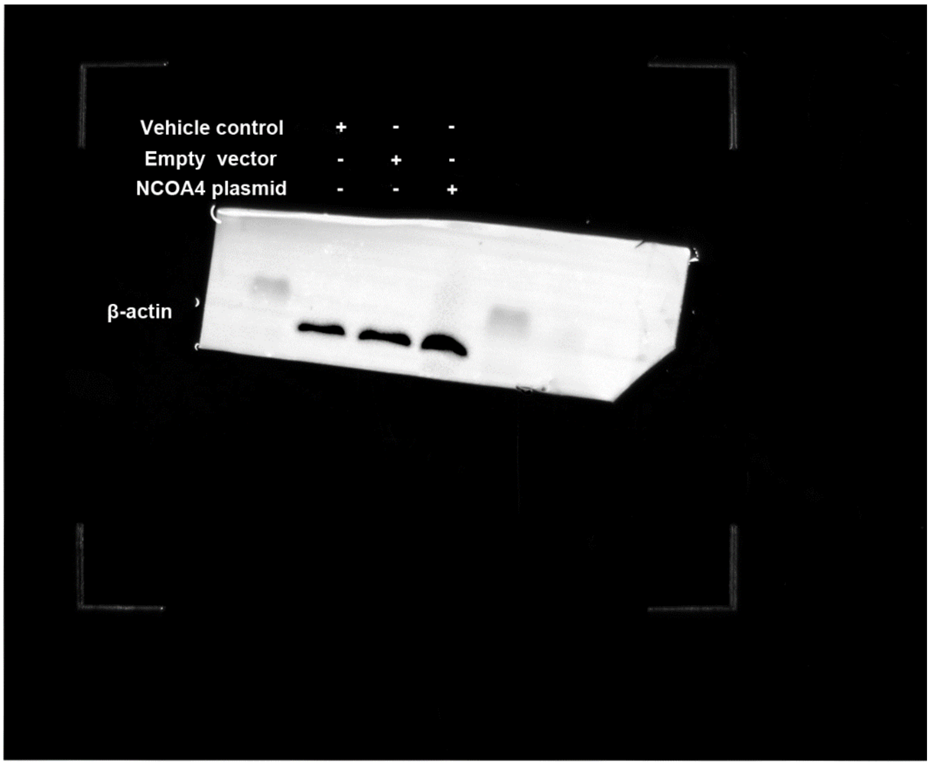

Supplementary Figure S4. Original western blot of Figure4 b.

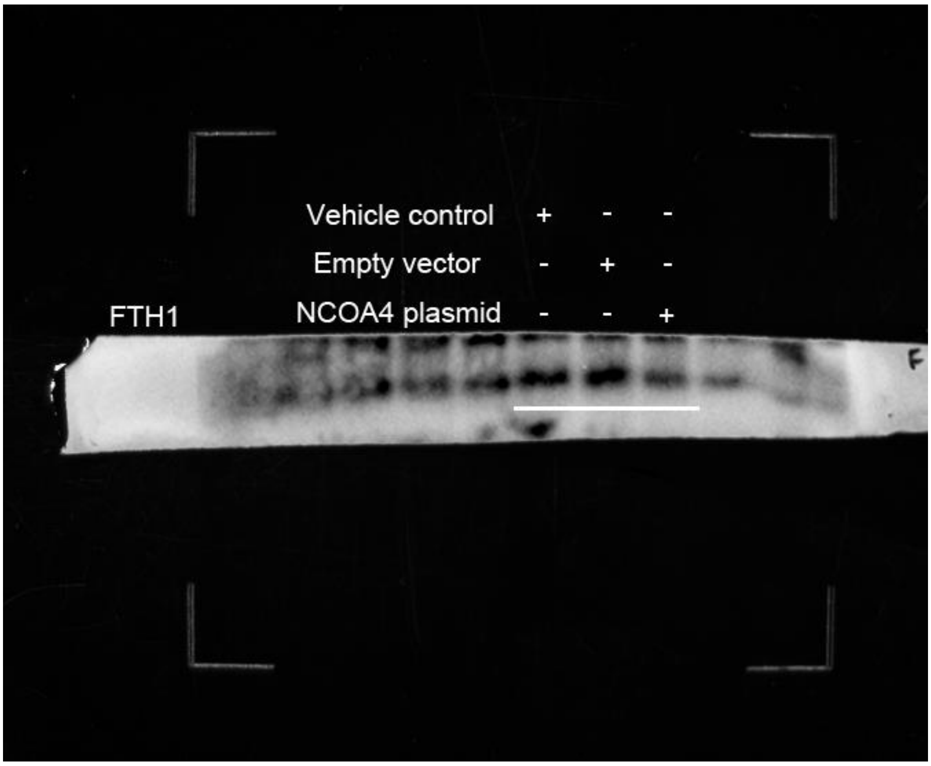

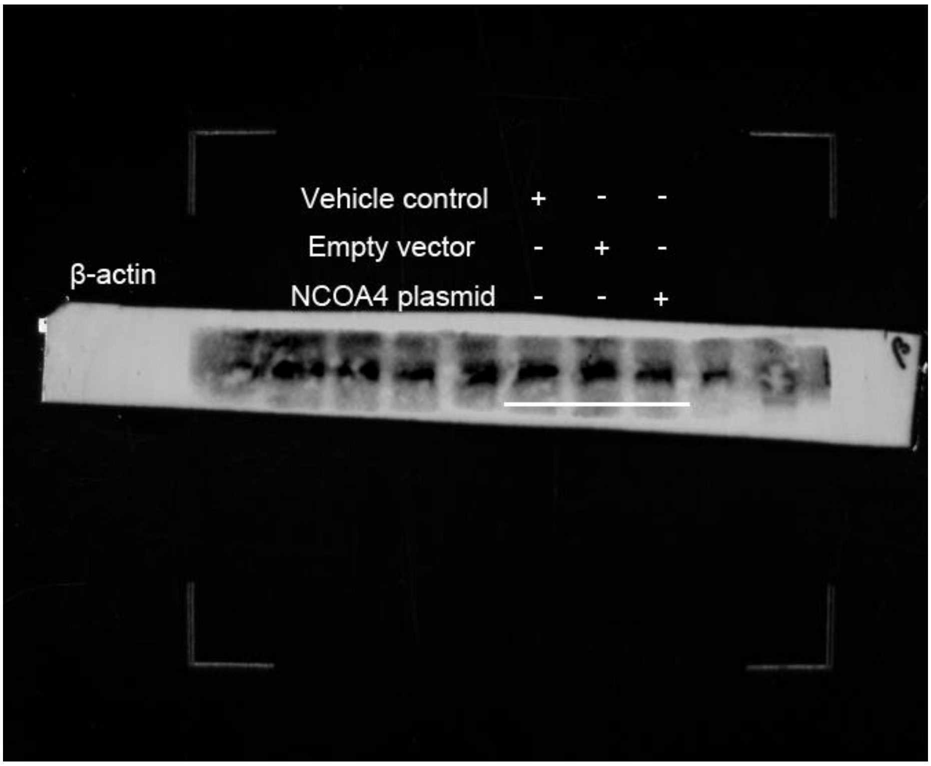

Supplementary Figure S5. Original western blot of Figure4 f.
